# The CXCR4-targeted theranostics era: a comprehensive review of a decade of progress (2015–2025)

**DOI:** 10.7150/thno.130266

**Published:** 2026-05-01

**Authors:** Biao Yang, Wenzhu Hu, Xiao Zhang, Yongkang Gai, Rui An, Chunxia Qin, Mengting Li, Xiaoli Lan

**Affiliations:** 1 Department of Nuclear Medicine, Union Hospital, Tongji Medical College, Huazhong University of Science and Technology, Wuhan 430022, China.; 2 Hubei Province Key Laboratory of Molecular Imaging, Wuhan 430022, China.; 3 Key Laboratory of Biological Targeted Therapy, the Ministry of Education, Wuhan 430022, China.

**Keywords:** CXCR4, theranostics, Pentixafor, positron emission tomography, targeted radionuclide therapy

## Abstract

Over the past decade, theranostics targeting the chemokine receptor CXCR4 have evolved from a promising concept into a clinical reality, revolutionizing the management of a diverse spectrum of diseases. This paradigm shift has been fueled by the development of [^68^Ga]Pentixafor, a high-affinity PET radiotracer that enables the precise, non-invasive visualization of CXCR4 expression *in vivo*. Its diagnostic prowess extends beyond conventional oncology, demonstrating superior performance to [^18^F]FDG in hematologic malignancies and offering critical decision-making insights for endocrine disorders and inflammatory conditions. Seamlessly completing the theranostic loop, the therapeutic counterparts [^177^Lu]/[^90^Y]Pentixather deliver targeted radiotherapy to CXCR4-expressing tissues, with pioneering applications in advanced multiple myeloma and acute myeloid leukemia establishing a compelling safety and efficacy profile. While challenges in target heterogeneity and toxicity persist, the future of CXCR4-targeted theranostics is bright, poised for advancement through combinatorial immunotherapies, alpha-emitting radionuclides, and artificial intelligence-driven patient stratification. This review comprehensively synthesizes a decade of progress, affirming CXCR4-targeted theranostics as a cornerstone of precision medicine that faithfully adheres to the "see what you treat, treat what you see" philosophy.

## 1. Introduction

Chemokine receptor CXCR4, a class A G protein-coupled receptor, mediates diverse physiological and pathological processes through its interaction with the endogenous ligand CXCL12 1,2. This signaling axis plays crucial roles in cancer progression, immune surveillance, and stem cell homing 3. Under physiological conditions, CXCR4 is constitutively expressed on T lymphocytes and macrophages, where it regulates lymphocyte trafficking and facilitates the migration, homing, and retention of hematopoietic stem cells within the bone marrow niche 4. Moreover, CXCR4 serves as a coreceptor for human immunodeficiency virus type 1 (HIV-1), collaborating with CD4 to enable viral entry into host T cells 5-7.

**Figure 1:**
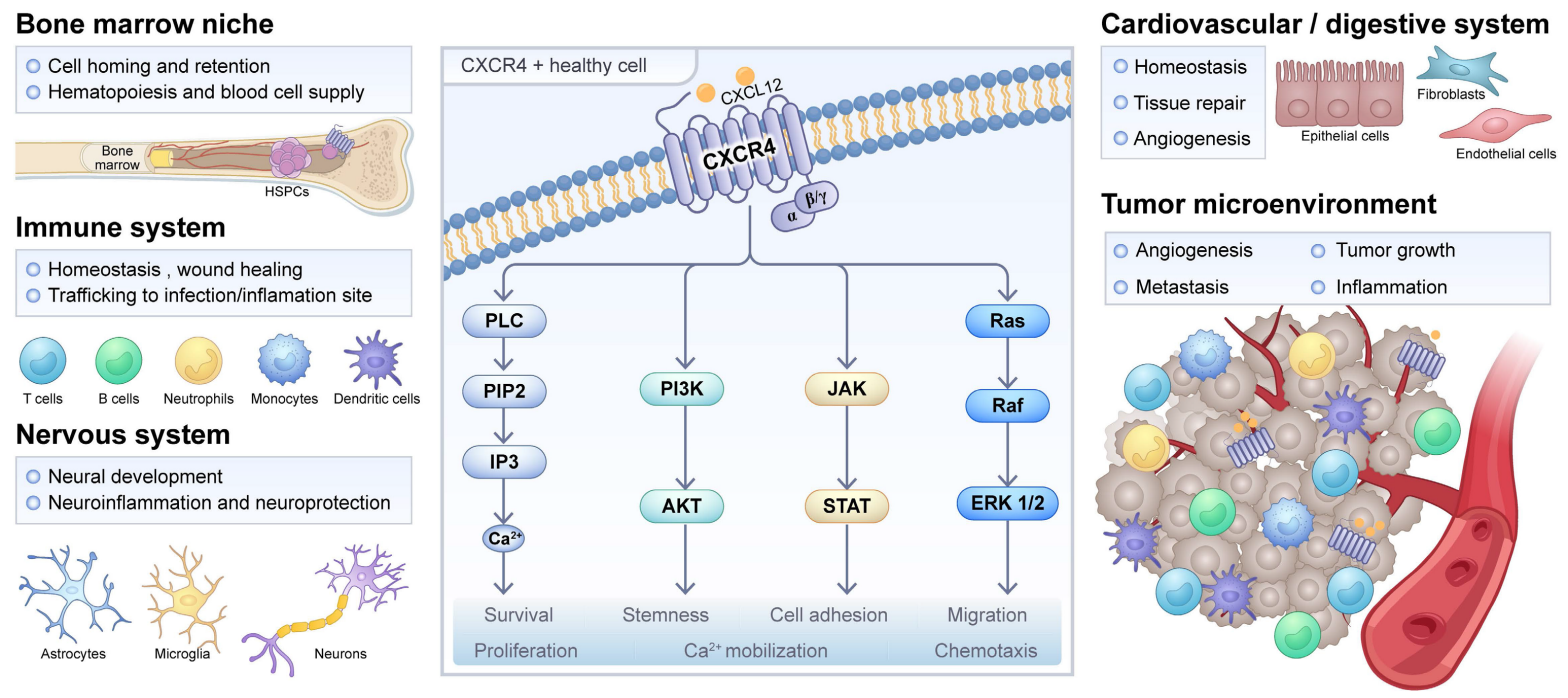
** CXCR4 signaling in physiological or pathological processes.** The physiological role of CXCR4 is listed for hematopoietic stem and progenitor cells (HSPCs) in the bone marrow and other cell types in the immune, nervous, cardiovascular, and digestive systems. Meanwhile, the pathological role of CXCR4 in inflammation, infection, injury repair, and the tumor microenvironment is also indicated. The CXCR4/CXCL12 interaction and the activated main signaling pathways, namely PLC, PI3K/Akt, JAK/STAT, and MAPK, are shown in the middle, along with their downstream cellular responses. Adapted with permission from 18, copyright © 2025, Springer Nature.

In oncological contexts, CXCR4 is frequently overexpressed in a wide spectrum of malignancies, including hematologic neoplasms such as multiple myeloma (MM) and acute myeloid leukemia (AML), as well as solid tumors like breast, prostate, and pancreatic cancers 8-12. Such dysregulated expression is closely associated with enhanced metastatic potential, therapeutic resistance, and unfavorable prognosis. Mechanistically, CXCR4-CXCL12 signaling promotes tumor cell survival, proliferation, and dissemination 13. Within the tumor microenvironment, CXCR4 is also expressed on various immune cells, including dendritic cells, regulatory T cells, and CD8^+^ T cells, where it participates in shaping immunosuppressive landscapes 14. Beyond oncology, this axis is implicated in the pathogenesis of inflammatory disorders, cardiovascular diseases, and infectious conditions, though the full scope of its molecular mechanisms remains to be fully elucidated 15-17. Compared to other G protein-coupled receptors, CXCR4 presents itself as a particularly compelling target for molecular theranostics. This is due to its frequent and marked overexpression in a wide range of diseases, its efficient ligand-induced internalization and its well-established role in driving key disease pathways such as metastasis, cell survival, and therapy resistance (Figure 1).

Owing to its central role in disease mechanisms, CXCR4 has garnered significant interest as a target for molecular imaging and targeted radionuclide therapy. A key milestone was achieved in 2015 with the development of [^68^Ga]Pentixafor at the Technical University of Munich 19. This radiotracer is based on a high-affinity cyclic pentapeptide scaffold (cyclo(D-Tyr^1^-[NMe]-D-Orn^2^-Arg^3^-2-Nal^4^-Gly^5^)) and enables non-invasive assessment of CXCR4 expression via positron emission tomography (PET). In an initial proof-of-concept human study involving four patients with lymphoproliferative diseases, [^68^Ga]Pentixafor PET demonstrated high imaging contrast and clinical feasibility, paving the way for broader translational applications. Subsequent efforts have focused on developing therapeutic counterparts such as ^177^Lu- and ^90^Y-labeled Pentixather, which are designed to deliver cytotoxic radiation to CXCR4-expressing tumors 20.

Notably, the field of CXCR4-targeted theranostics has expanded to include a broader range of ligand development. For example, the literature has reported novel CXCR4 antagonists with higher affinity, whose innovative design strategies and promising preclinical profiles pave the way for next-generation radiopharmaceuticals 21,22. Meanwhile, clinical research is no longer limited to Pentixafor. Recent studies have summarized early clinical results of alternative CXCR4-targeted tracers based on different scaffolds 23,24. Collectively, these advances demonstrate that CXCR4 imaging has evolved into a versatile platform with multiple tracer options, each potentially possessing unique pharmacokinetic or binding properties. In this review, we summarize key preclinical advances in the development of CXCR4-directed radiopharmaceuticals and highlight emerging clinical applications of [^68^Ga]Pentixafor for diagnostic imaging and [^177^Lu]/[^90^Y]Pentixather for endoradiotherapy in malignant and selected benign diseases.

## 2. Preclinical studies of CXCR4-targeted radiopharmaceuticals

Given the critical role of CXCR4 in mediating cancer metastasis, it was identified as a promising therapeutic target as early as 2011 25. A novel high-affinity cyclic CXCR4 ligand, [^68^Ga]Pentixafor (cyclo(D-Tyr^1^-[NMe]-D-Orn^2^-[4-(aminomethyl)benzoic acid,^68^Ga-DOTA]-Arg^3^-2-Nal^4^-Gly^5^)), was developed and systematically validated both *in vitro* and *in vivo* using nude mouse models bearing metastatic OH1 human small cell lung cancer (SCLC) xenografts. This radiotracer demonstrated exceptional *in vivo* stability, highly specific tumor uptake, and minimal off-target accumulation in non-tumor tissues, thereby establishing a solid foundation for the development of CXCR4-targeted radiopharmaceuticals. Subsequent research has evaluated derivatives labeled with alternative radiometals (e.g., AMD- and T140-based analogs) across various tumor models 19,26-28. A growing body of evidence supports [^68^Ga]Pentixafor PET imaging as a highly translational approach for assessing CXCR4 expression *in vivo*, both in preclinical models and clinical cancer populations 29-32. This diagnostic advancement has facilitated the parallel development of corresponding therapeutic agents—notably [^177^Lu]/[^90^Y]Pentixather—enabling integrated theranostic strategies for personalized CXCR4-directed therapy.

The efficacy of [^68^Ga]Pentixafor as a leading CXCR4-targeted imaging probe has been rigorously validated through comprehensive preclinical evaluations across multiple cancer models. Wester *et al.* employed two lymphoma models, Daudi (a human Burkitt's lymphoma cell line with high CXCR4 expression) and SU-DHL-8 (a human large B-cell lymphoma cell line with low CXCR4 expression), to demonstrate that [^68^Ga]Pentixafor exhibits high affinity and selectivity for human CXCR4, along with favorable pharmacokinetic properties 19. In MM research, NOD SCID mice xenografted with MM.1S and OPM-2 cells underwent sequential [^18^F]FDG and [^68^Ga]Pentixafor PET imaging 32. Notably, [^68^Ga]Pentixafor achieved significantly higher mean tumor-to-background ratios (TBRs) in both xenograft models compared to [^18^F]FDG, highlighting its superior diagnostic performance. Additionally, [^68^Ga]Pentixafor PET imaging enabled precise visualization of heterogeneous CXCR4 expression levels in tumor xenografts derived from patient-derived xenograft models of T-cell acute lymphoblastic leukemia and AML 33. Researchers have further employed *in vitro* cell-binding assays and the chorioallantoic membrane xenograft model to evaluate the diagnostic potential of [^68^Ga]Pentixafor in colorectal cancer imaging, expanding its preclinical applications across both hematologic malignancies and solid tumors 34.

Beyond its established applications in cancer models, the noninvasive diagnostic potential of [^68^Ga]Pentixafor has been extensively investigated in inflammatory diseases, including atherosclerosis, heart failure, and acute myocardial infarction (AMI). A preclinical study in experimental rabbit models demonstrated the tracer's capability to detect CXCR4 expression on inflammatory cells within atherosclerotic plaques 30. Compared to normal control arteries, significantly elevated radiotracer uptake was observed in atherosclerotic lesions of the abdominal aorta (mean TBR = 1.95 ± 0.51 *vs*. 1.22 ± 0.25; *p* < 0.05) and right carotid artery (mean TBR = 1.24 ± 0.38 *vs*. 0.96 ± 0.37; *p* < 0.05). Autoradiographic analysis confirmed that radiotracer accumulation in plaque vessel walls was predominantly localized to macrophage-rich regions, with uptake intensity showing a strong positive correlation with CXCR4 expression levels in corresponding histological sections. Furthermore, [^68^Ga]Pentixafor PET imaging successfully visualized leukocyte infiltration in post-myocardial infarction hearts and detected acute, diffuse myocardial inflammatory cell infiltration in a murine model of pressure overload-induced heart failure (transverse aortic constriction) 35. These findings collectively establish [^68^Ga]Pentixafor as a highly specific, noninvasive PET biomarker for CXCR4 expression in inflammatory lesions, enabling quantitative assessment of spatiotemporal inflammatory cell dynamics, a crucial parameter for monitoring disease progression, therapeutic response, and stratifying patients for anti-inflammatory interventions.

## 3. Clinical applications of diagnostic radiopharmaceutical [^68^Ga]Pentixafor

Based on the aforementioned principles, the clinical applications of the diagnostic radiopharmaceutical [^68^Ga]Pentixafor have diversified significantly, demonstrating substantial value across numerous domains including endocrine disorders, hematologic malignancies, solid tumors, and inflammatory diseases. By enabling non-invasive quantification of CXCR4 receptor expression *in vivo*, this imaging modality provides crucial molecular imaging evidence for precise diagnosis, subtype differentiation, staging, prognostic evaluation, and treatment decision-making in the aforementioned conditions. The following sections will provide a systematic review and detailed discussion of these extensive applications (Table 1).

### 3.1. Endocrine disorders

#### 3.1.1. Primary aldosteronism (PA)

PA represents the most prevalent cause of secondary hypertension, characterized by autonomous excessive aldosterone secretion from the adrenal cortex 124. Aldosterone-producing adenoma (APA) and idiopathic hyperaldosteronism (IHA) constitute the two main PA subtypes, accounting for approximately 35% and 60% of cases, respectively. Subtype differentiation, particularly distinguishing between APA and IHA, remains a crucial diagnostic challenge in clinical practice, as it directly influences therapeutic strategy selection (e.g., adrenalectomy versus mineralocorticoid receptor antagonist therapy) and long-term cardiovascular/renal outcomes 125,126.

**Figure 2:**
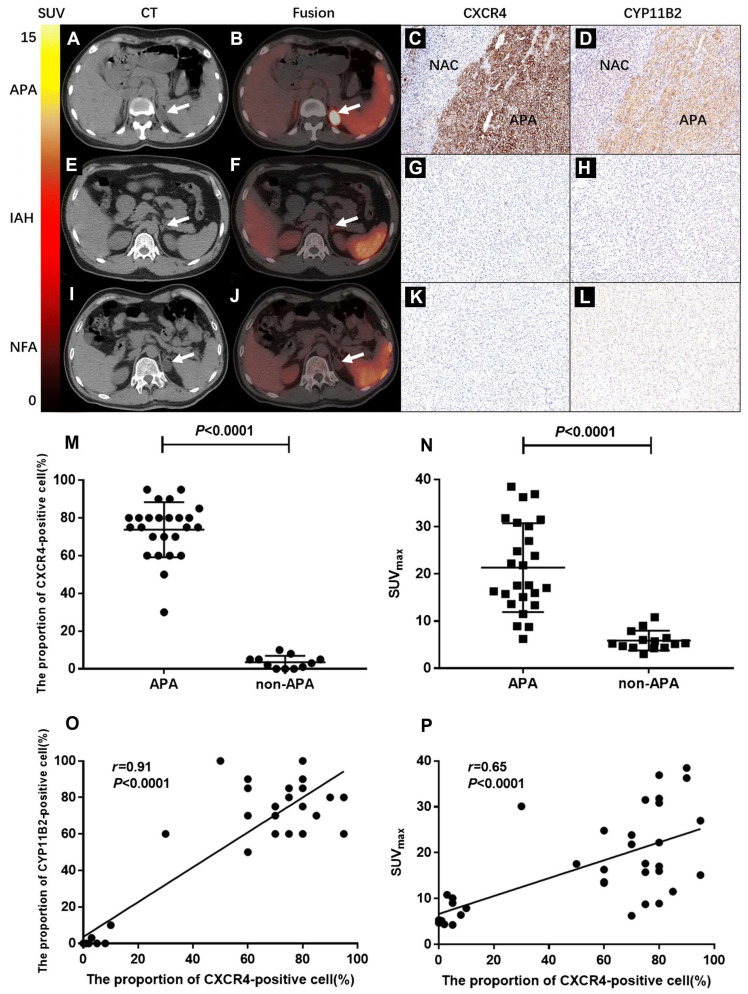
** Clinical application of [^68^Ga]Pentixafor in PA**. **(A, B)** Typical [^68^Ga]Pentixafor PET/CT images of a 44-year-old female. She had fatigue, hypertension, and serum potassium at 2.2 mmol/L. True-positive uptake (SUVmax 36.4, white arrow) was seen in an APA lesion in the left adrenal gland, with strong CXCR4 and CYP11B2 expression (**C, D**; NAC: normal adrenal cortex). CT showed a 2×2.3 cm left adrenal lesion with significant uptake. Post-surgery, her blood pressure and potassium normalized. **(E, F)** Typical images of a 26-year-old male. The test was true-negative in an IAH case. His lowest potassium was 2.7 mmol/L. CT revealed a 1.2 cm left adrenal nodule with slightly increased uptake (SUVmax = 5.3). Immunohistochemistry showed no increased CXCR4 and CYP11B2 expression **(G, H)**. Postoperative hypertension persisted. **(I, J)** Typical images: a 44-year-old male's NFA lesion was true-negative. CT showed a 2.2 cm hypodense left adrenal nodule with no increased uptake (SUVmax = 5.24, white arrow). Histopathology confirmed an adenoma. Immunohistochemistry showed no increased CXCR4 and CYP11B2 expression **(K, L)**. **(M)** The proportion of CXCR4-positive cells (%) was significantly higher in APA than in non-APA lesions (*p* < 0.0001). **(N)** SUVmax values were significantly elevated in APA lesions (21.34 ± 9.41) compared to non-APA lesions (6.29 ± 2.10; *p* < 0.0001). **(O)** Scatter plot demonstrates a significant correlation between CXCR4 expression and CYP11B2 expression levels (%) (*r* = 0.91, *p* < 0.0001). **(P)** A significant correlation was observed between SUVmax of [^68^Ga]Pentixafor PET and CXCR4 expression levels (*r* = 0.65, *p* < 0.0001). Adapted with permission from 61, copyright © 2020, Springer Nature.

Recent investigations have revealed that CXCR4 is highly expressed on APA cell membranes and demonstrates a significant positive correlation with aldosterone synthase (CYP11B2) expression levels, while showing minimal expression in non-functioning adrenal adenomas (NFA) 127. This molecular characteristic establishes CXCR4-specific radiotracer [^68^Ga]Pentixafor as a convenient, intuitive, and clinically valuable tool for PA subtype classification through targeted molecular imaging. In a prospective study by Jie Ding *et al.*, 36 patients with clinically suspected PA underwent [^68^Ga]Pentixafor PET/computed tomography (CT) imaging followed by unilateral adrenalectomy 61. Histopathological and clinical evaluation identified 39 adrenal lesions, including 25 APA, 4 IHA, and 10 NFA. Visual assessment demonstrated that [^68^Ga]Pentixafor PET/CT achieved 100% sensitivity, 78.6% specificity, and 92.3% accuracy in discriminating APA (Figure 2A-I). The maximum standardized uptake value (SUVmax) in APA lesions (21.34 ± 9.41) significantly exceeded that in non-APA lesions (6.29 ± 2.10; *p* < 0.0001) (Figure 2M-N). These findings establish [^68^Ga]Pentixafor PET/CT as a reliable non-invasive modality for APA detection (Figure 2O-P). A subsequent study by the same research group demonstrated that [^68^Ga]Pentixafor PET/CT achieved detection rates exceeding 90% for functional adrenal nodules, with particularly superior performance for nodules ≥ 1 cm in diameter. Owing to its high sensitivity and specificity, this imaging technique shows promise as a valuable tool for surgical decision-making in PA management.

Recent comparative studies have evaluated the diagnostic performance of [^68^Ga]Pentixafor PET/CT against adrenal venous sampling for subtyping and lateralization of aldosterone hypersecretion. Results indicated over 80% concordance between the two modalities for lateralization in PA patients, including those with bilateral adrenal hyperplasia 108. In summary, substantial evidence supports the clinical utility of [^68^Ga]Pentixafor PET/CT across multiple aspects of PA management, including subtype classification, identification of APA/nodules, functional lateralization, therapeutic decision-making, and prognostic evaluation.

#### 3.1.2. Cushing syndrome (CS)

CS represents an endocrine disorder characterized by chronic exposure to excessive cortisol levels, broadly categorized into adrenocorticotropic hormone (ACTH)-dependent (70%-80%) and ACTH-independent (20%-30%) forms 128. ACTH-dependent CS primarily results from ACTH-producing pituitary adenomas (Cushing's disease) or ectopic ACTH-secreting lesions, while ACTH-independent CS typically stems from autonomous cortisol-secreting adrenal adenomas/carcinomas or, more rarely, bilateral macronodular adrenal hyperplasia 129.

**Figure 3:**
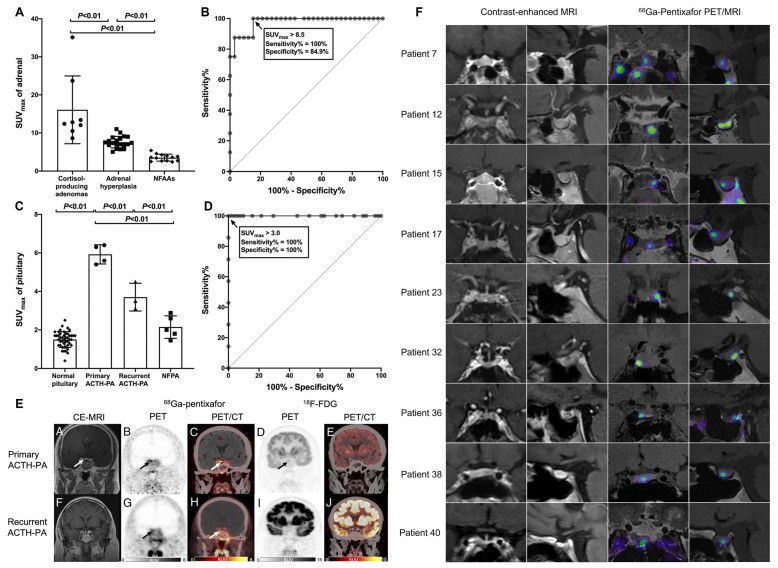
** Clinical application of [^68^Ga]Pentixafor in CS**. **(A)** SUVmax comparison of [^68^Ga]Pentixafor in cortisol-producing adenoma, adrenal hyperplasia, and nonfunctioning adrenal adenoma. **(B)** ROC analysis of [^68^Ga]Pentixafor SUVmax for discriminating cortisol-producing adenoma from adrenal hyperplasia and nonfunctioning adrenal adenoma. **(C)** SUVmax distribution of [^68^Ga]Pentixafor across normal pituitary, primary/recurrent ACTH-producing pituitary adenoma, and nonfunctioning pituitary adenoma. **(D)** ROC evaluation of [^68^Ga]Pentixafor SUVmax for differentiating ACTH-producing pituitary adenoma from nonfunctioning pituitary adenoma and normal pituitary. **(E)** A 41-year-old man with Cushing's disease, pathologically confirmed as a right-sided ACTH-producing pituitary adenoma (5 mm maximum diameter). Contrast-enhanced MRI demonstrated a hypointense microadenoma in the right pituitary wing (arrow). [^68^Ga]Pentixafor PET/CT coronal PET and fusion images revealed focal uptake (SUVmax 6.3; arrows), while [^18^F]FDG PET/CT showed only mild diffuse pituitary uptake without distinct foci (arrows). A 41-year-old woman with recurrent right-sided ACTH-producing pituitary adenoma (3 mm maximum diameter). Contrast-enhanced MRI was negative. [^68^Ga]Pentixafor coronal PET and fusion images demonstrated increased uptake (SUVmax 4.5; arrows), whereas [^18^F]FDG coronal PET and fusion images showed no abnormal uptake in the pituitary fossa. Adapted with permission from 81, copyright © 2022, Wolters Kluwer.** (F)** Coronal and sagittal contrast-enhanced MRI and corresponding [^68^Ga]Pentixafor PET/MRI scans in study participants show examples of negative cases (no detected lesion). Adapted with permission from 105, copyright © 2024, Radiological Society of North America.

Emerging evidence indicates that CXCR4 is highly expressed in both ACTH-secreting tumors and adrenal cortical lesions. This molecular characteristic has positioned [^68^Ga]Pentixafor PET imaging as an increasingly valuable tool for differential diagnosis across CS subtypes 130. In a retrospective analysis by Jie Ding *et al.*, 31 patients (16 with CS and 15 with non-functioning pituitary or adrenal adenomas) underwent [^68^Ga]Pentixafor PET/CT imaging 81. Eleven pituitary adenoma patients additionally received [^18^F]FDG PET/CT for comparison. The study established that using an SUVmax threshold of >8.5 for adrenal lesions, [^68^Ga]Pentixafor PET/CT achieved 100% sensitivity and 84.9% specificity in diagnosing cortisol-producing adenomas. When applying an SUVmax cutoff of 3.0 for pituitary lesions, the technique demonstrated perfect discrimination (100% sensitivity and specificity) for identifying ACTH-producing pituitary adenomas (Figure 3A-D). Notably, significant correlations were observed between [^68^Ga]Pentixafor SUVmax and biochemical parameters in pituitary adenomas, including serum ACTH (*r* = 0.71), serum cortisol (*r* = 0.80), and 24-hour urinary free cortisol (*r* = 0.53; all *p* < 0.05). Importantly, no such correlations were found with glucose metabolism parameters, underscoring the specificity of CXCR4 expression in these endocrine tumors (Figure 3E).

A subsequent prospective study involving 43 Cushing's disease patients further demonstrated that [^68^Ga]Pentixafor PET/magnetic resonance imaging (MRI) outperformed contrast-enhanced MRI alone in localizing ACTH-secreting pituitary adenomas, highlighting the pivotal role of this multimodal molecular imaging approach in preoperative tumor localization for Cushing's disease management 105 (Figure 3F).

### 3.2. Hematological malignancies

#### 3.2.1. MM

MM is characterized by clonal plasma cell proliferation in the bone marrow and overproduction of monoclonal immunoglobulins 131. CXCR4 activation has been implicated in MM-related bone disease, while the CXCR4/CXCL12 axis promotes plasma cell proliferation. The CXCR4-targeted PET tracer [^68^Ga]Pentixafor has recently been applied in MM evaluation 31. Biodistribution studies demonstrate favorable dosimetry for [^68^Ga]Pentixafor, with an effective dose of 2.3 mSv per 150 MBq administration. The bladder wall receives the highest absorbed dose (12.2 mGy), followed by the spleen (8.1 mGy) and kidneys (5.3 mGy), all lower than corresponding doses from [^18^F]FDG or ^68^Ga-labeled somatostatin receptor ligands.

**Figure 4:**
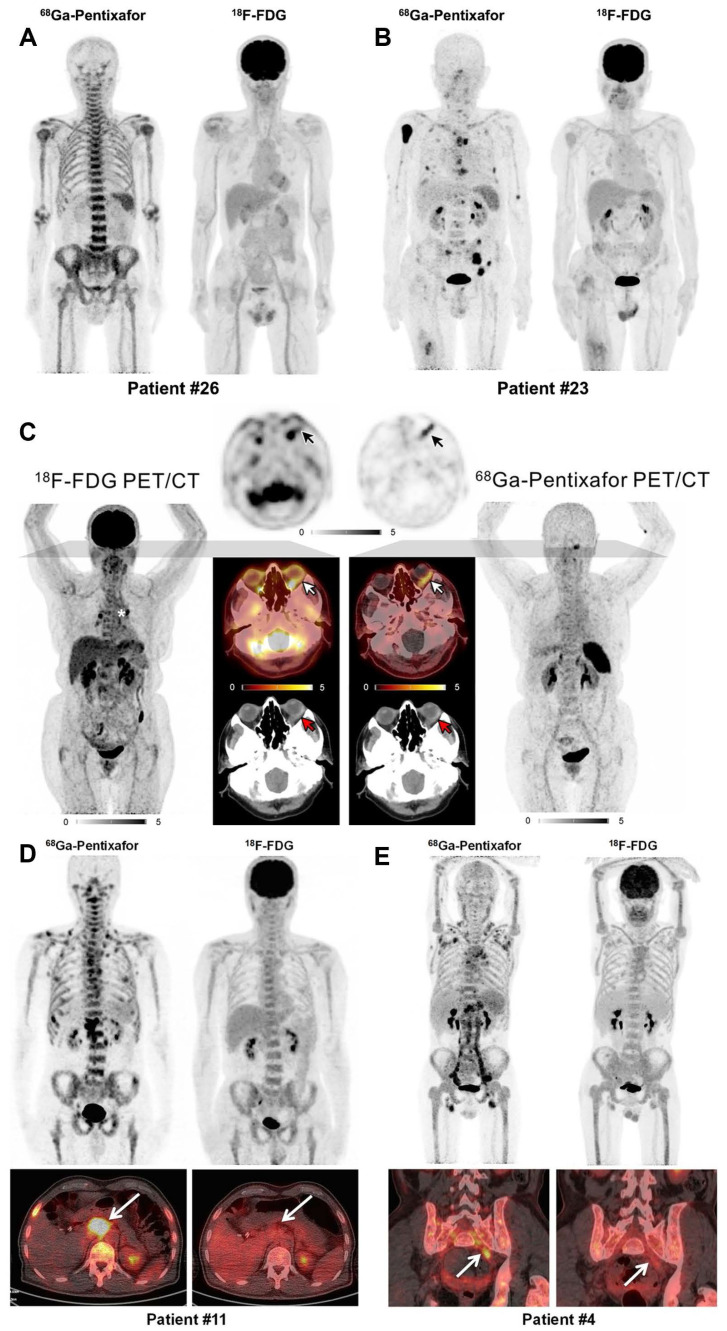
** Clinical application of [^68^Ga]Pentixafor in MM, MZL, and WM/LPL. (A)** Patient #26 with IgA-λ MM demonstrating diffuse bone marrow infiltration pattern. Intense [^68^Ga]Pentixafor uptake in bone marrow with negative [^18^F]FDG avidity. **(B)** Patient #23 with IgA-λ MM. Multiple bone marrow lesions detected by [^68^Ga]Pentixafor PET, while [^18^F]FDG uptake remained negative. Adapted with permission from 60, copyright © 2020, Springer Nature.** (C)** Maximum-intensity-projection images of [^18^F]FDG and [^68^Ga]Pentixafor PET in a patient with EMZL. Central axial sections demonstrate orbital lymphoma manifestation with discordant tracer uptake ([^18^F]FDG-negative, CXCR4-positive), annotated with white (PET/CT), black (PET), and red (CT) arrows. Asterisk indicates intense focal uptake in two hilar lymph nodes; biopsy confirmed sarcoidosis, not MZL. Adapted with permission from 68, copyright © 2021, Society of Nuclear Medicine and Molecular Imaging. **(D)** Patient 11 with WM (IgM λ), ISS-WM score 2 (indeterminate risk). [^68^Ga]Pentixafor imaging demonstrated intense bone marrow uptake with multifocal lesions and CXCR4-positive lymph nodes (arrows). [^18^F]FDG activity (score 4) showed homogeneous bone marrow distribution without nodal avidity (arrows). **(E)** Patient 4 with WM (IgM κ) and Bing-Neel syndrome, ISS-WM score 2 (indeterminate risk). Multiple CXCR4-positive lymph nodes were detected in cervical, axillary, hepatoduodenal, retroperitoneal, iliac, and inguinal regions, most undetected by [^18^F]FDG PET. The involved left iliac nerve root (arrow) was CXCR4-positive but [^18^F]FDG-negative. Adapted with permission from 55, copyright © 2019, Society of Nuclear Medicine and Molecular Imaging.

While [^18^F]FDG PET/CT plays a role in MM workup, its accuracy is limited by false-negative results due to hexokinase-2 expression loss in MM cells, and false-positive findings from fractures, inflammatory changes, or MM-related anemia. A prospective cohort study by Pan *et al.* compared [^68^Ga]Pentixafor and [^18^F]FDG PET/CT in newly diagnosed MM patients (*n* = 30) 60 (Figure 4A-B). [^68^Ga]Pentixafor demonstrated significantly higher detection rates (93.3% *vs*. 53.3%, *p* = 0.0005), particularly in cases with diffuse bone marrow patterns (88.2% *vs*. 29.4%, *p* = 0.002). Quantitative parameters of [^68^Ga]Pentixafor uptake (TBmUCXCR4, SUVmax, SUVmean) showed significant positive correlations with end-organ damage severity, disease stage, and tumor burden biomarkers including serum β2-microglobulin, serum free light chains, and 24-hour urinary light chains. These findings underscore the prognostic value of [^68^Ga]Pentixafor PET/CT in MM, supported by multiple investigations 72,83. Emerging evidence suggests splenic uptake of [^68^Ga]Pentixafor may provide prognostic information in pretreated MM patients, comparable to diffusion-weighted MRI 85. In comparative studies, [^68^Ga]Pentixafor PET demonstrated superior or equal sensitivity versus [^18^F]FDG in 63% of cases 43. [^68^Ga]Pentixafor PET demonstrates detectable CXCR4 expression across a broad spectrum of MM subtypes, suggesting its potential as a widely applicable imaging biomarker. However, the intensity of uptake (e.g., SUVmax) can vary and has been correlated with disease burden and stage, indicating its quantitative value for risk stratification.

#### 3.2.2. Marginal zone lymphoma (MZL)

MZL, accounting for approximately 7% of indolent non-Hodgkin lymphomas, includes extranodal (EMZL), nodal (NMZL), and splenic (SMZL) subtypes 132. CXCR4 is physiologically expressed on lymphocytes and has been observed in various T-cell and B-cell neoplasms, including MZL 89. [^68^Ga]Pentixafor PET has shown promising results in MZL assessment. A pilot study in treatment-naïve lymphoma patients demonstrated that [^68^Ga]Pentixafor PET/CT significantly improved MZL detection compared to conventional staging on both per-patient and per-lesion bases (*p* < 0.001) 68 (Figure 4C). Among 18 PET-guided biopsies, 16 confirmed MZL involvement. These findings highlight the utility of [^68^Ga]Pentixafor PET in primary MZL staging, corroborated by subsequent CXCR4-directed PET/MRI studies 57. In newly diagnosed MZL patients, [^68^Ga]Pentixafor identified more disease sites than [^18^F]FDG across all subtypes 94. Subgroup analyses further indicated superior diagnostic performance of [^68^Ga]Pentixafor PET/CT for NMZL and EMZL, suggesting its potential as a preferred novel PET agent for MZL evaluation.

#### 3.2.3. Waldenström macroglobulinemia/lymphoplasmacytic lymphoma (WM/LPL)

WM/LPL is a rare indolent NHL characterized by bone marrow lymphoplasmacytic infiltration and monoclonal immunoglobulin production 71. While [^18^F]FDG PET/CT is valuable for staging FDG-avid nodal lymphomas, its utility in WM/LPL is limited except when assessing aggressive transformation 133. Studies demonstrate elevated CXCR4 expression in WM/LPL B-cells compared to healthy donors, prompting evaluation of [^68^Ga]Pentixafor PET/CT in this malignancy 134,135. A prospective cohort study (*n* = 17) revealed significantly higher detection rates with [^68^Ga]Pentixafor versus [^18^F]FDG PET/CT (100% *vs*. 58.8%; *p* = 0.023) 55. For bone marrow involvement, sensitivities were 94.1% and 58.8%, respectively (*p* = 0.077), while for nodal involvement, [^68^Ga]Pentixafor demonstrated markedly superior detection (76.5% *vs*. 11.8%; *p* = 0.003) (Figure 4D-E). Additionally, [^68^Ga]Pentixafor identified more paraspinal and central nervous system lesions than [^18^F]FDG.

Further investigation evaluated [^68^Ga]Pentixafor PET/CT for treatment response assessment in WM/LPL (*n* = 15) 71. All patients showed positive baseline [^68^Ga]Pentixafor PET/CT, compared to only 11 with [^18^F]FDG PET/CT. Following chemotherapy (overall response rate: 86.7%), [^68^Ga]Pentixafor PET/CT accurately identified tumor responses in all cases, including new lesions or significantly increased uptake in two progressing patients. In contrast, [^18^F]FDG PET/CT failed to detect improvement in 6/13 responding patients and missed progression in 1/2 progressing cases. [^68^Ga]Pentixafor PET/CT outperforms [^18^F]FDG in uptake intensity, bone marrow involvement visualization, focal lesion detection, and treatment response assessment in WM/LPL.

#### 3.2.4. Mantle cell lymphoma (MCL)

**Figure 5:**
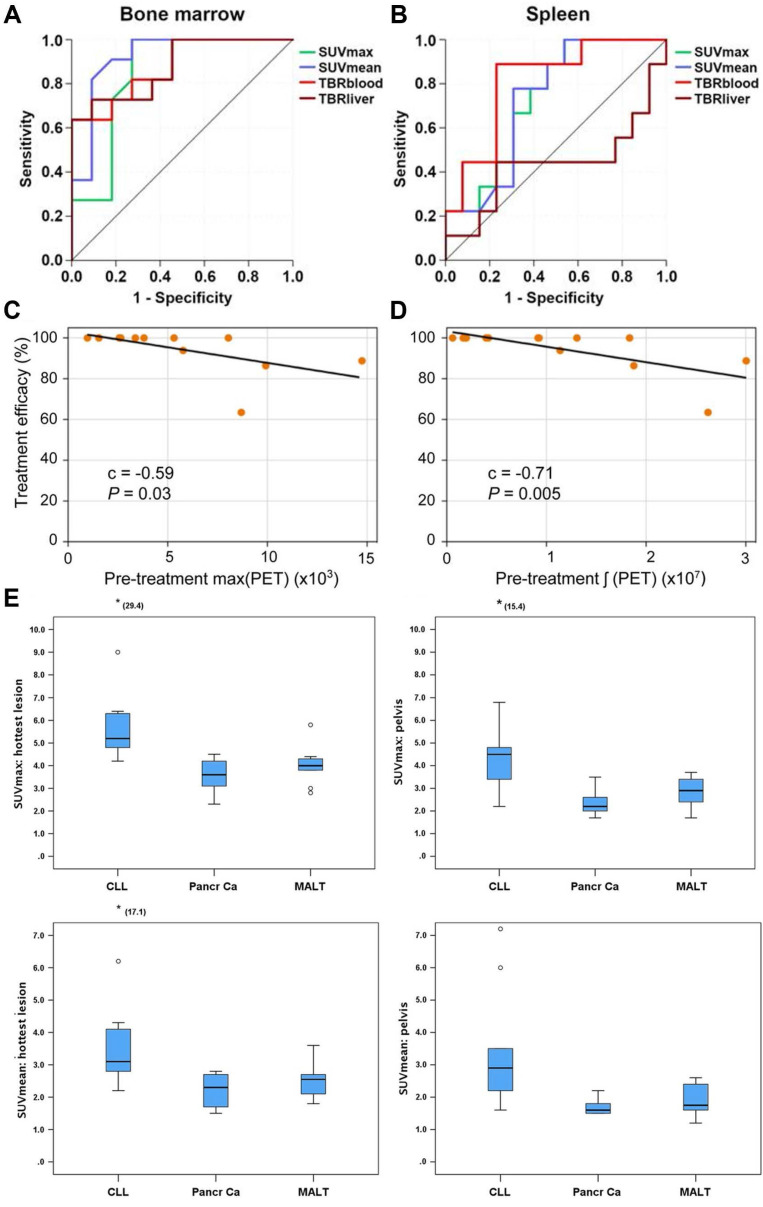
** Clinical application of [^68^Ga]Pentixafor in MCL, CNSL, and CLL.** Receiver operating characteristic curves demonstrating the diagnostic performance of [^68^Ga]Pentixafor PET SUV and TBR for detecting bone marrow **(A)** and splenic involvement **(B)** in MCL patients. Adapted with permission from 66, copyright © 2021, Ivyspring International Publisher. Lesion-based analysis in CNSL patients (14 lymphoma lesions): Correlations between treatment response metric (η) and pretreatment PET parameters—max(PET) **(C)**, and ∫(PET) **(D)**. Scatter plots show Pearson correlation coefficient** (C)** and *P* values, with black line indicating least-squares regression. Adapted with permission from 62, copyright © 2020, Society of Nuclear Medicine and Molecular Imaging. **(E)** Box plots demonstrating differences in SUVmax and SUVmean within pelvic bone marrow and the hottest bone marrow lesions among patients with CLL, pancreatic adenocarcinoma, and MALT lymphoma. Adapted with permission from 51, copyright © 2018, Wolters Kluwer.

MCL, an aggressive B-cell NHL with approximately 50% 5-year survival, commonly involves lymph nodes, spleen, bone marrow, and gastrointestinal tract 66,78,136,137. Although [^18^F]FDG PET is recommended for MCL staging, its uptake can be low to moderate and unreliable for bone marrow assessment 138,139. A prospective study comparing [^68^Ga]Pentixafor and [^18^F]FDG in MCL patients (*n* = 22) demonstrated significantly higher sensitivity for [^68^Ga]Pentixafor PET (100% *vs*. 75.2%, *p* < 0.001), with superior SUVs and TBRs 66. For bone marrow involvement (biopsy-confirmed), [^68^Ga]Pentixafor SUVmean showed an AUC of 0.92, while for splenic involvement, TBRblood achieved an AUC of 0.81. [^68^Ga]Pentixafor PET shows promise for non-invasive assessment of bone marrow and splenic involvement in MCL, with significantly higher detection rates and better tumor-to-background contrast than [^18^F]FDG (Figure 5A-B). In treatment response evaluation, [^68^Ga]Pentixafor PET detected complete remission earlier and more comprehensively than MRI 88. At interim assessment, 56.3% of target lesions met CR criteria by [^68^Ga]Pentixafor PET versus 50.0% by MRI (*p* = 0.63); at end-of-treatment, corresponding rates were 70.2% versus 47.4% (*p* = 0.021), indicating superior performance of [^68^Ga]Pentixafor PET in evaluating treatment response, particularly upon therapy completion.

#### 3.2.5. Central nervous system lymphoma (CNSL)

A proof-of-concept study evaluated [^68^Ga]Pentixafor PET in 11 CNSL patients (8 PCNSL, 3 SCNSL) 62. Quantitative analysis in 7 pretreatment patients revealed excellent tumor-to-brain parenchyma contrast (TBR > 5:1) in all active disease cases. Initial CXCR4 uptake levels significantly correlated with subsequent treatment response to regimens like high-dose methotrexate (*p* < 0.05), with higher CXCR4 expression associated with better remission rates (Figure 5C-D).

#### 3.2.6. Chronic lymphocytic leukemia (CLL)

A prospective study first reported [^68^Ga]Pentixafor application in CLL, comparing 13 CLL patients with 20 controls (10 pancreatic cancer, 10 mucosa-associated lymphoid tissue, MALT lymphoma) 51 (Figure 5E). [^68^Ga]Pentixafor uptake was significantly higher in CLL bone marrow than in tumor patients without marrow involvement. Notably, nodal lesion uptake exceeded bone marrow uptake, consistent with previous reports of higher proliferation rates in lymph nodes versus bone marrow in CLL 140.

### 3.3. Solid tumors

#### 3.3.1. Glioblastoma (GBM)

GBM, representing 45-50% of primary malignant brain tumors in adults, demonstrates poor prognosis due to its infiltrative growth pattern, frequent recurrence, and therapeutic resistance 141,142. CXCR4 overexpression correlates with tumor angiogenesis and adverse outcomes in GBM 143,144. The clinical application of CXCR4-targeted [^68^Ga]Pentixafor PET/CT has been increasingly explored in this context.

**Figure 6:**
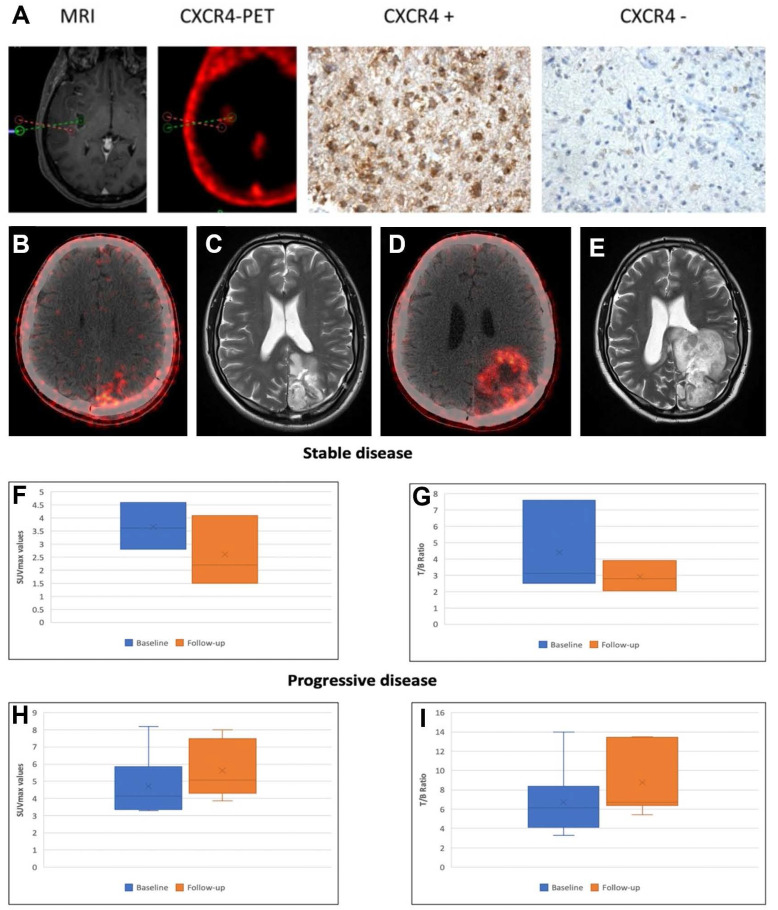
** Clinical application of [^68^Ga]Pentixafor in GBM. (A)** CXCR4 Expression in PET-Positive *vs*. PET-Negative Tumor Samples (assessed by [^68^Ga]Pentixafor PET). Representative immunohistochemical staining showing CXCR4 expression in [^68^Ga]Pentixafor-negative (red dotted area; [^18^F]FET-positive) and positive (green) tumor regions. Integrated neuronavigation with [^68^Ga]Pentixafor PET/CT and MP-RAGE MRI guided targeted biopsy. H&E nuclear staining. Magnification: 200×. Adapted with permission from 40, copyright © 2016, Ivyspring International Publisher. Axial fused [^68^Ga]Pentixafor PET/CT images in a 31-year-old male GBM patient post-craniotomy demonstrate tracer uptake (SUVmax = 8.18, T/B ratio = 6.5) in a 2.0 cm left parietal lobe lesion **(B)**. 3-month post-R-CT follow-up [^68^Ga]Pentixafor PET/CT **(D)** indicates disease progression (SUVmax = 7.3, T/B ratio = 13.5). Corresponding T2-weighted axial MRI at baseline **(C)** and 3-month post-treatment **(E)** confirm disease progression with increased tumor size. Box-and-whisker plots showing decreased SUVmax **(F)** and T/B ratios **(G)** in patients with stable disease, and increased SUVmax **(H)** and T/B ratios **(I)** in those with progressive disease. Adapted with permission from 97, copyright © 2024, Wolters Kluwer.

A preoperative imaging study enrolled 15 patients with suspected primary or recurrent GBM 40. [^68^Ga]Pentixafor PET/CT demonstrated visual positivity in 13/15 cases, with SUVmean 3.0 ± 1.5 and SUVmax 3.9 ± 2.0. While absolute uptake values were lower than those of [^18^F]FET PET/CT (SUVmean 4.4 ± 2.0, SUVmax 5.3 ± 2.3), [^68^Ga]Pentixafor achieved significantly higher TBRs for both SUVmean (154.0 ± 90.7 *vs*. 4.1 ± 1.3) and SUVmax (70.3 ± 44.0 *vs*. 3.8 ± 1.2, *p* < 0.01). Histopathological analysis confirmed concordance between high [^68^Ga]Pentixafor uptake regions and CXCR4 expression, establishing the feasibility of non-invasive CXCR4 imaging in GBM (Figure 6A). Analysis of the GEO dataset GSE16011 (*n* = 284) revealed negligible CXCR4 mRNA expression in healthy brain tissue versus significant inter- and intra-tumoral heterogeneity in GBM, supporting [^68^Ga]Pentixafor's potential for patient stratification in CXCR4-directed therapies 74.

In treatment response assessment, Waheed *et al.* performed serial [^68^Ga]Pentixafor PET/CT in 10 GBM patients undergoing radiochemotherapy (R-CT) 97. Baseline mean SUVmax (4.6 ± 2.1) showed no significant difference from post-R-CT values (4.4 ± 1.6). However, during 6-month follow-up, patients with stable disease (*n* = 4) exhibited significantly lower SUVmax and tumor/blood ratios (3.70 ± 0.90, 2.64 ± 1.35) compared to baseline (4.40 ± 2.8, 2.91 ± 0.93), while progressing patients (*n* = 6) demonstrated significantly elevated parameters versus both baseline and stable disease groups (*p* < 0.05) (Figure 6B-I). These findings suggest [^68^Ga]Pentixafor's potential for R-CT response assessment, though larger multicenter trials are warranted for validation.

#### 3.3.2. Head and neck squamous cell carcinoma (HNSCC)

HNSCC, comprising over 90% of head and neck malignancies, ranks as the sixth most common cancer globally with poor overall survival 145,146. While [^18^F]FDG remains the primary radiotracer for HNSCC staging, its limitations include poor detection of small tumors and difficulty distinguishing recurrence from post-treatment changes.

**Figure 7:**
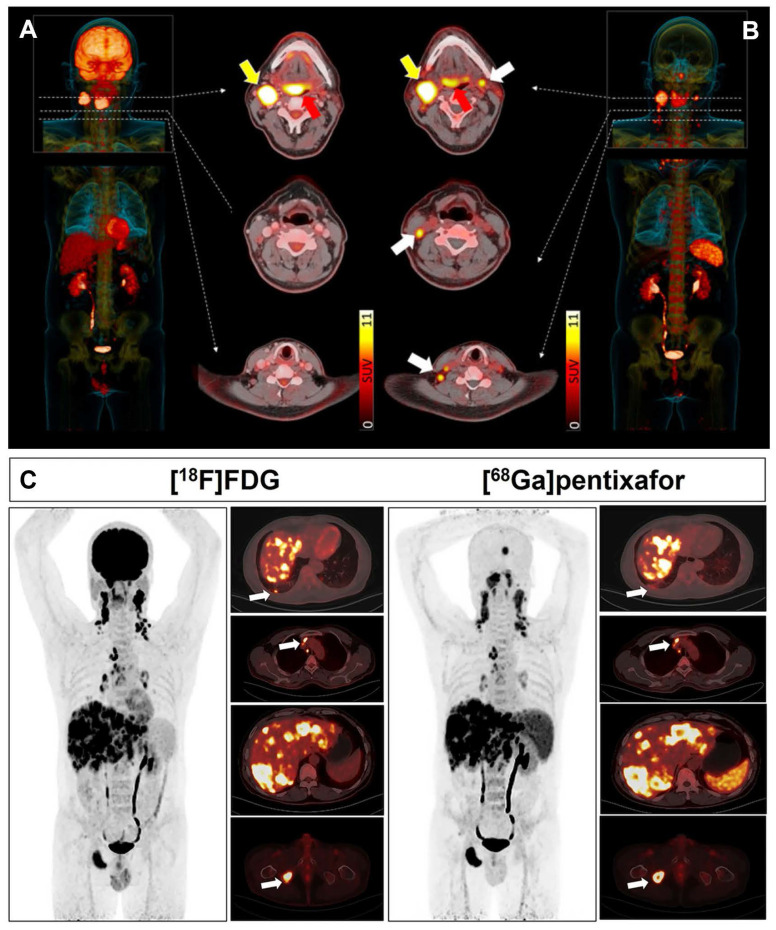
** Clinical application of [^68^Ga]Pentixafor in HNSCC. (A, B)** Patient with tongue base carcinoma (red arrow). [^18^F]FDG PET/CT shows a histologically confirmed right-sided lymph node metastasis (yellow arrow). Additional cervical lymph node findings (white arrows) were exclusively detected by [^68^Ga]Pentixafor PET/CT. Left: [^18^F]FDG PET/CT MIP. Right: [^68^Ga]Pentixafor PET/CT MIP. Middle: Three transaxial PET/CT slices for [^18^F]FDG (left) and [^68^Ga]Pentixafor (right). While the primary tumor showed higher [^18^F]FDG than [^68^Ga]Pentixafor uptake (red arrow), [^18^F]FDG identified a single metastatic LN at the right jaw angle (yellow arrow), histologically confirmed. [^68^Ga]Pentixafor PET/CT detected multiple CXCR4-positive LNs along bilateral cervical neurovascular sheaths, initially suspected as metastases. Histology confirmed these LNs (white arrows) represented inflammatory/reactive changes with lymphofollicular hyperplasia. Adapted with permission from 93, copyright © 2023, e-Century Publishing Corporation. **(C)** Comparative [^18^F]FDG and [^68^Ga]Pentixafor imaging in a 49-year-old treatment-naïve male with NPC and multiorgan metastases. Both PET/CT scans demonstrate abnormal tracer uptake in: lung (arrow; SUVmax 5.20 vs 3.00), mediastinal lymph nodes (arrow; SUVmax 9.75 vs 7.35), liver (SUVmax 11.00 vs 10.01), and bone (arrow; SUVmax 8.48 vs 11.57). Adapted with permission from 99, copyright © 2024, Springer Nature.

Comparative studies by Zhi *et al.* demonstrated lower detection rates for [^68^Ga]Pentixafor versus [^18^F]FDG in HNSCC, particularly for primary tumors 93 (Figure 7A-B). Immunohistochemistry revealed variable CXCR4 expression in both primary tumors and lymph nodes, with no significant correlation between *in vitro* CXCR4 upregulation and [^68^Ga]Pentixafor parameters (TBR: *r* = 0.33, *p* = 0.39; SUVmax: *r* = 0.44, *p* = 0.2). Notably, CXCR4 expression in germinal center centroblasts sometimes exceeded tumor cell expression, suggesting inflammatory microenvironment contributions 147. The limited performance of [^68^Ga]Pentixafor may be attributed to reactive lymphoid hyperplasia and immune cell infiltration within the tumor microenvironment 148.

Subtype analysis revealed more pronounced tracer accumulation in nasopharyngeal carcinoma (NPC) compared to oropharyngeal and oral cavity malignancies 102. A prospective study demonstrated comparable detection rates between [^68^Ga]Pentixafor and [^18^F]FDG PET/CT for primary nasopharyngeal tumors and metastatic cervical lymph nodes 99 (Figure 7C). A distinct advantage of [^68^Ga]Pentixafor is the absence of physiological uptake in brown adipose tissue, eliminating potential imaging interference in cervical regions.

#### 3.3.3. Lung malignancies

SCLC, representing 13-15% of lung cancers, demonstrates early hematogenous spread to brain, liver, and bones, with 60-70% of patients presenting with extensive-stage disease at diagnosis 149,150. Initial studies documented non-invasive CXCR4 assessment in SCLC using [^68^Ga]Pentixafor PET, demonstrating positivity in 8/10 patients with superior lesion detection compared to [^68^Ga]DOTATOC PET and significantly higher TBRs 39. While [^18^F]FDG detected two cases missed by [^68^Ga]Pentixafor, the latter identified equal or greater numbers of lesions in remaining patients, supporting its utility for detecting CXCR4 expression and screening patients for targeted therapies.

In non-small cell lung cancer (NSCLC), [^68^Ga]Pentixafor PET/CT demonstrated remarkable clarity in detecting brain metastases, addressing a known limitation of [^18^F]FDG PET 151. Comprehensive analysis across lung cancer subtypes revealed significantly higher [^68^Ga]Pentixafor uptake in SCLC (mean SUVmax = 10.3 ± 5.0) compared to NSCLC (*n* = 75) and pulmonary neuroendocrine neoplasms (*n* = 5, *p* = 0.005) 87. Among NSCLC subtypes, adenocarcinoma (*n* = 16) showed significantly higher uptake (mean SUVmax = 8.0 ± 1.9) than squamous cell carcinoma (*n*=54; 6.2 ± 2.1) and NOS subtypes (*n* = 5; 5.8 ± 1.5, *p* = 0.003). [^68^Ga]Pentixafor PET/CT demonstrated good sensitivity (85.7%) and specificity (78.1%) in differentiating SCLC from NSCLC at ROC cutoff SUVmax 7.2, and similar performance (sensitivity 87.5%, specificity 71.4%) in distinguishing adenocarcinoma from squamous cell carcinoma at cutoff SUVmax 6.7.

#### 3.3.4. Other cancer types

**Figure 8:**
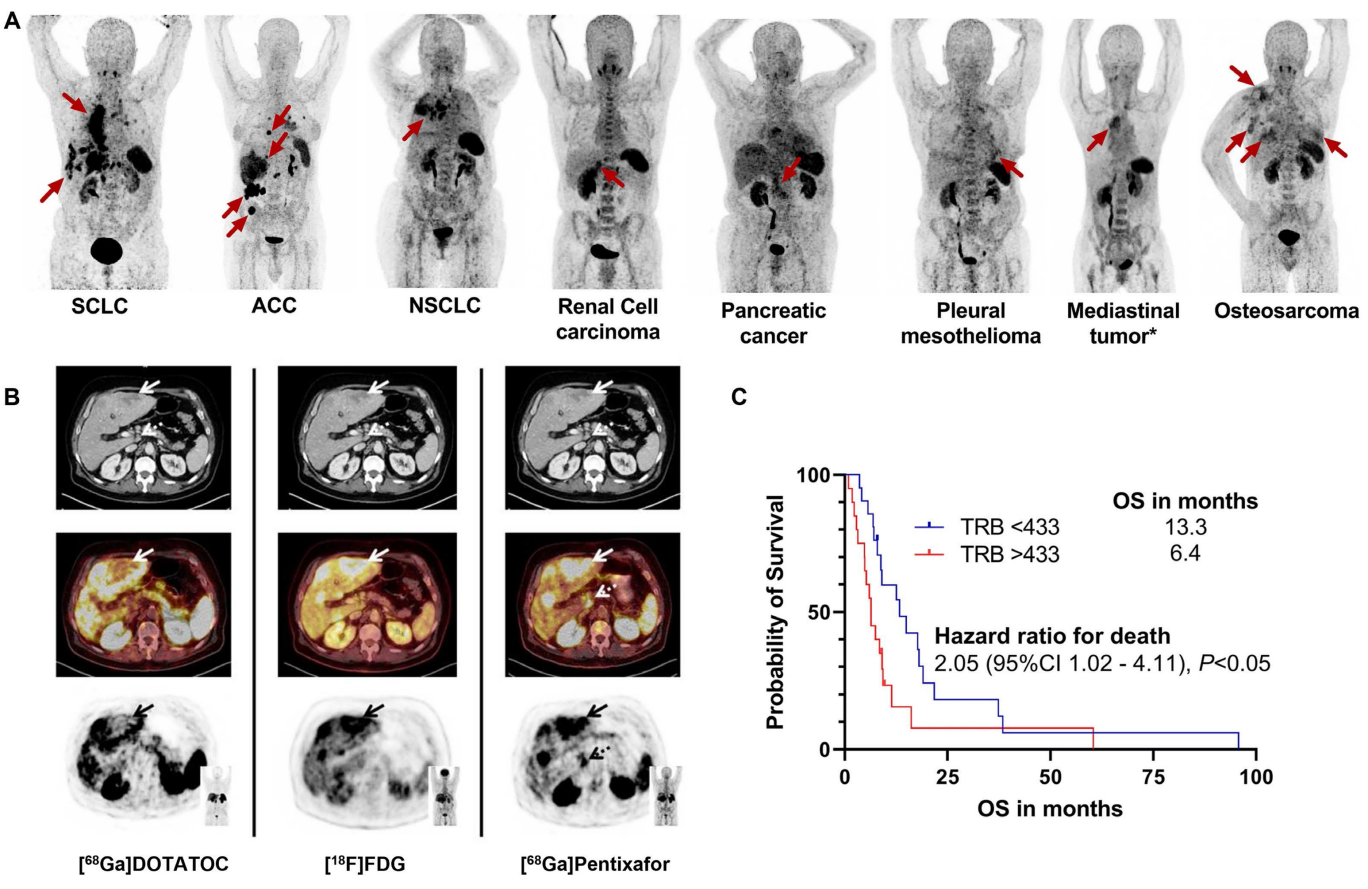
** Clinical application of [^68^Ga]Pentixafor in other cancer types. (A)** CXCR4-targeted PET/CT with [^68^Ga]Pentixafor across solid tumor entities. Maximum intensity projections are displayed. Red arrows indicate CXCR4-positive tumor lesions. ACC: adrenocortical carcinoma; NEN: neuroendocrine neoplasia; NSCLC: non-small cell lung carcinoma; SCLC: small cell lung carcinoma. *Not otherwise specified. Adapted with permission from 95, copyright © 2024, Springer Nature. **(B)** Tumor heterogeneity in a G3 gastric NET with liver metastases (Ki67: 90%). Hypermetabolic liver lesions show SSTR loss and CXCR4 upregulation (solid arrows; [^68^Ga]Pentixafor SUVmax 10.3 *vs* [^68^Ga]DOTATOC SUVmax 3.8). [^68^Ga]Pentixafor exclusively detected a suspicious celiac lymph node (dotted arrow), providing additional staging information. All transaxial PET/(CT) images displayed at window level 0-5.5. Adapted with permission from 45, copyright © 2017, Ivyspring International Publisher. **(C)** Kaplan-Meier analysis of overall survival by tumor receptor binding (TRB) on [^68^Ga]Pentixafor PET. Elevated TRB (stratified by median value) correlates with reduced survival. Adapted with permission from 101, copyright © 2024, Springer Nature.

[^68^Ga]Pentixafor PET has been extensively evaluated in various malignancies including pancreatic, prostate, breast, hepatocellular carcinoma, sarcomas, and cancers of unknown primary 38. A comprehensive study involving 142 patients with 23 different histologically confirmed solid tumors undergoing 152 [^68^Ga]Pentixafor PET/CT scans demonstrated exceptional image contrast with median TBR >4 across all tumor types compared to normal physiological background 95 (Figure 8A). These data not only confirm the widespread overexpression of CXCR4 across various solid tumors but, more importantly, their high-contrast imaging characteristics provide crucial visual evidence for accurately identifying CXCR4-positive lesions and guiding subsequent targeted therapies.

Notably, substantial variations in [^68^Ga]Pentixafor positivity rates exist among different tumor types, suggesting that transcriptomic profiling or whole-cell protein measurements may not fully reflect actual cell surface CXCR4 expression 1,152. This discrepancy may stem from differences in receptor internalization and recycling regulated by the tumor microenvironment, variations in the activation status of downstream signaling pathways, or uneven subclonal distribution due to tumor heterogeneity. Therefore, *in vivo* PET imaging may hold greater clinical relevance than *ex vivo* molecular assays in assessing functional, targetable CXCR4 expression. In gastrointestinal and pancreatic neuroendocrine tumors (GEP-NETs), [^68^Ga]Pentixafor positivity correlated with high proliferative activity (Ki67 index ≥ 85%), while well-differentiated tumors showed minimal receptor expression 45 (Figure 8B). All CXCR4-positive subjects demonstrated high [^18^F]FDG uptake but relatively low or absent somatostatin receptor expression as evaluated by [^68^Ga]DOTATOC, indicating an inverse correlation between CXCR4 and SSTR2 expression with increasing tumor grade in G1-G3 neuroendocrine tumors 153. This finding carries significant implications for subtyping and treatment guidance. CXCR4 imaging may help identify more aggressive subsets of neuroendocrine tumors that are less responsive to traditional somatostatin analog therapies, thereby steering treatment toward more intensive strategies.

In adrenal cortical carcinoma (ACC), *in vitro* experiments confirmed high CXCR4 expression at lesion sites. [^68^Ga]Pentixafor PET TBR independently correlated with shorter overall survival in metastasized ACC patients, providing valuable prognostic information for disease progression monitoring and treatment guidance 101 (Figure 8C). This suggests that CXCR4 imaging is not merely a diagnostic tool but may also serve as a biomarker for predicting aggressive tumor biological behavior and patient prognosis. Future research could further explore incorporating quantitative PET parameters (such as SUVmax, TBR) into risk stratification models to optimize therapeutic decision-making.

While these findings are encouraging, it is essential to recognize the challenges before CXCR4-targeted theranostics can be widely applied in solid tumors. These include intra- and inter-tumoral heterogeneity, physiological uptake of tracers in non-target tissues, and the complex biological barriers involved in translating high diagnostic sensitivity into effective therapeutic responses. Future research should focus on identifying patient subpopulations most likely to benefit from CXCR4-targeted therapies and exploring combination strategies with other treatment modalities, such as immunotherapy.

### 3.4. Inflammatory diseases

The chemokine receptor CXCR4 demonstrates significant expression across various inflammatory pathologies, playing crucial roles in immune cell recruitment and chronic inflammation maintenance 30,154. In atherosclerosis, the CXCL12/CXCR4 axis exerts pro-atherogenic, pro-thrombotic, and plaque-destabilizing effects. CXCR4 expression is observed not only in monocytes migrating to arterial lesions, differentiated macrophages, and lymphocytes, but also in smooth muscle progenitor cells and endothelial progenitor cells participating in plaque evolution. Accumulating evidence from both experimental and clinical studies supports the utility of [^68^Ga]Pentixafor PET for detecting CXCR4 expression levels in vascular walls 30,49.

**Figure 9:**
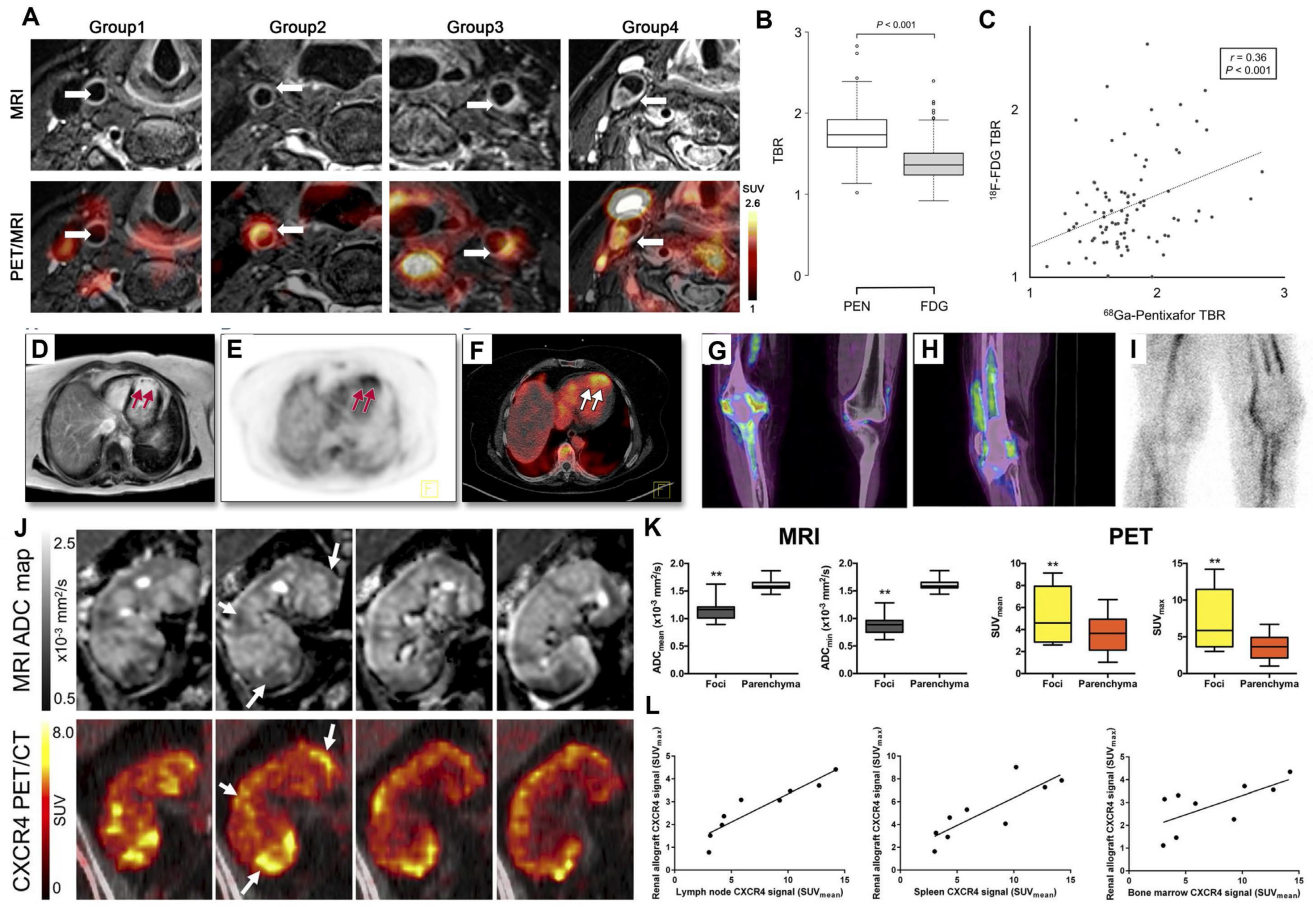
** Clinical application of [^68^Ga]Pentixafor in inflammatory diseases. (A)** Representative transaxial [^68^Ga]Pentixafor PET/MRI images of carotid lesions across study groups. Focal tracer uptake is seen in mildly atherosclerotic carotids with slight eccentric thickening (Group 1), and in moderately (Group 3) to severely (Group 4) atherosclerotic carotids with marked eccentric thickening. No significant uptake is observed in control carotids without eccentric thickening (Group 1). Arrows indicate arterial regions of interest. Adapted with permission from 54, copyright © 2019, Springer Nature. Lesion-based comparison of [^68^Ga]Pentixafor and [^18^F]FDG uptake in atherosclerotic plaques. Box plot **(B)** and scatter plot **(C)** demonstrate correlation between [^18^F]FDG and [^68^Ga]Pentixafor (PEN) uptake measured by TBR. Adapted with permission from 59, copyright © 2020, Society of Nuclear Medicine and Molecular Imaging. Increased [^68^Ga]Pentixafor uptake in acute myocardial infarction involving the left anterior descending artery. Axial views of **(D)** contrast-enhanced multishot IR-TFE CMR, **(E)** CXCR4-targeted PET, and **(F)** fused PET/CT demonstrate apical tracer uptake colocalizing with myocardial damage on CMR (arrows). Adapted with permission from 155, copyright © 2015, ELSEVIER. Definite osteomyelitis in a 52-year-old patient with elevated CRP 5 months after total knee arthroplasty removal and spacer implantation. Coronal **(G)** and sagittal **(H)** [^68^Ga]Pentixafor PET/CT show increased tracer uptake in tibial and femoral bones (including bone marrow). **(I)** Sagittal [^99m^Tc]besilesomab scintigraphy at 4 h demonstrates osteitis without bone marrow uptake. Adapted with permission from 48, copyright © 2018, Society of Nuclear Medicine and Molecular Imaging.** (J)** Representative MR and PET images of acute renal allograft infection. ADC maps (left, coronal views) demonstrate reduced values colocalizing with CXCR4-upregulated foci on [^68^Ga]Pentixafor PET (right, arrows). T2-weighted MRI and MIP PET provide anatomical orientation. The renal allograft is located in the right lower abdomen. Spleen shows physiological CXCR4 expression due to leukocyte abundance. **(K)** Box plots of MRI ADC values and [^68^Ga]Pentixafor SUV in CXCR4-positive foci versus unaffected allograft parenchyma (*n* = 9). **(L)** CXCR4^+^ leukocyte accumulation coincides with bone marrow and lymphoid organ upregulation, indicating systemic inflammatory response. Boxes represent IQR (median line); whiskers show range. **p < 0.01. Adapted with permission from 44, copyright © 2017, Society of Nuclear Medicine and Molecular Imaging.

In a prospective study by Xiang Li *et al.*, 72 lymphoma patients underwent whole-body [^68^Ga]Pentixafor PET/MRI with dedicated T2-weighted carotid sequencing 54 (Figure 9A). Consistent with previous investigations, eccentric atherosclerotic lesions demonstrated significantly higher [^68^Ga]Pentixafor uptake compared to non-stenotic lesions, indicating elevated CXCR4 expression 30,49. Malte Kircher *et al.* directly compared [^68^Ga]Pentixafor PET/CT and [^18^F]FDG PET/CT for atherosclerosis imaging in large arterial walls 59. [^68^Ga]Pentixafor PET detected more lesions (*n* = 290; TBR ≥ 1.6, *p* < 0.01) with significantly higher uptake values (1.8 ± 0.5 *vs*. 1.4 ± 0.4; *p* < 0.01) than [^18^F]FDG PET (Figure 9B-C). However, only weak correlation was observed between the uptake patterns of these two tracers, necessitating further investigation to elucidate the biological mechanisms underlying CXCR4 positivity.

In AMI, CXCR4 facilitates stem cell and progenitor cell recruitment to infarct zones while regulating post-infarction inflammatory responses and resolution. The inaugural *in vivo* study of CXCR4 imaging in human hearts following myocardial infarction demonstrated enhanced chemokine expression in 3 of 7 patients with (sub)acute infarction 155 (Figure 9D-F). Tracer uptake in infarcted myocardium was two-fold higher than in remote myocardium, with negative results in the remaining four patients. Notably, troponin and creatine kinase levels were elevated in PET-positive compared to PET-negative patients, suggesting that hypoxia-induced myocardial damage may trigger CXCR4 upregulation to initiate healing processes. Subsequent research revealed time-dependent [^68^Ga]Pentixafor uptake in infarcted myocardium, showing linear decline before day 13 post-ischemia, indicating infiltrating inflammatory cells as the primary cellular source of tracer uptake 37,156.

Beyond atherosclerosis and myocardial infarction, CXCR4-targeted [^68^Ga]Pentixafor PET has been applied for non-invasive detection and localization of various inflammatory conditions including urinary tract infections, chronic skeletal infections, osteomyelitis, inflammatory bowel disease, and systemic sclerosis-associated interstitial lung disease 17,44,48,53,104. For chronic skeletal infections, a retrospective analysis of 14 patients undergoing [^68^Ga]Pentixafor PET/CT demonstrated positive results in 9 cases, with target-to-background ratios ranging from 5.1 to 15 (mean 8.7) 48 (Figure 9G-I). Pathological, bacteriological, or clinical confirmation identified 8 true positives among these 9 cases, with one false positive where MRI revealed vertebral necrotic plasmacytoma rather than spondylitis. These findings indicate [^68^Ga]Pentixafor PET/CT's suitability for diagnosing chronic skeletal infections.

Thorsten Derlin *et al.* compared spatial distribution and intensity of CXCR4 upregulation (via [^68^Ga]Pentixafor PET SUVs) with diffusion restriction (via MRI apparent diffusion coefficients) in 13 kidney transplant recipients with complicated urinary tract infections 44 (Figure 9J-L). PET-identified CXCR4 upregulation regions correlated with leukocyte infiltration areas showing increased cell density on MRI, supporting [^68^Ga]Pentixafor PET's potential for non-invasive leukocyte detection in renal allografts.

## 4. Clinical applications of therapeutic radiopharmaceutical [^177^Lu]/[^90^Y]Pentixather

Radiotheranostics represents an innovative and rapidly advancing paradigm in precision medicine that integrates diagnostic imaging with targeted radiotherapy 157,158. This approach fundamentally operates on the principle of delivering the right radiopharmaceutical to the right patient, optimizing therapeutic efficacy while minimizing adverse effects 159,160. By utilizing diagnostic imaging to identify suitable candidates and guide treatment planning, radiotheranostics enables precise lesion targeting and personalized dose delivery 161,162. This strategy proves particularly valuable for patients with disseminated or inoperable metastatic malignancies, offering new avenues for disease management. Established clinical examples include [^68^Ga]/[^177^Lu]DOTATATE for neuroendocrine tumors and [^68^Ga]/[^177^Lu]PSMA for prostate cancer 163-166. Building upon promising diagnostic results with the CXCR4-targeted radioligand [^68^Ga]Pentixafor, therapeutic analogs [^177^Lu]/[^90^Y]Pentixather have been developed, forming a novel theranostic pair for CXCR4-directed endoradiotherapy 105,119,123 (Table 2).

### 4.1. Clinical application status of [^177^Lu]/[^90^Y]Pentixather in various malignancies

**Figure 10:**
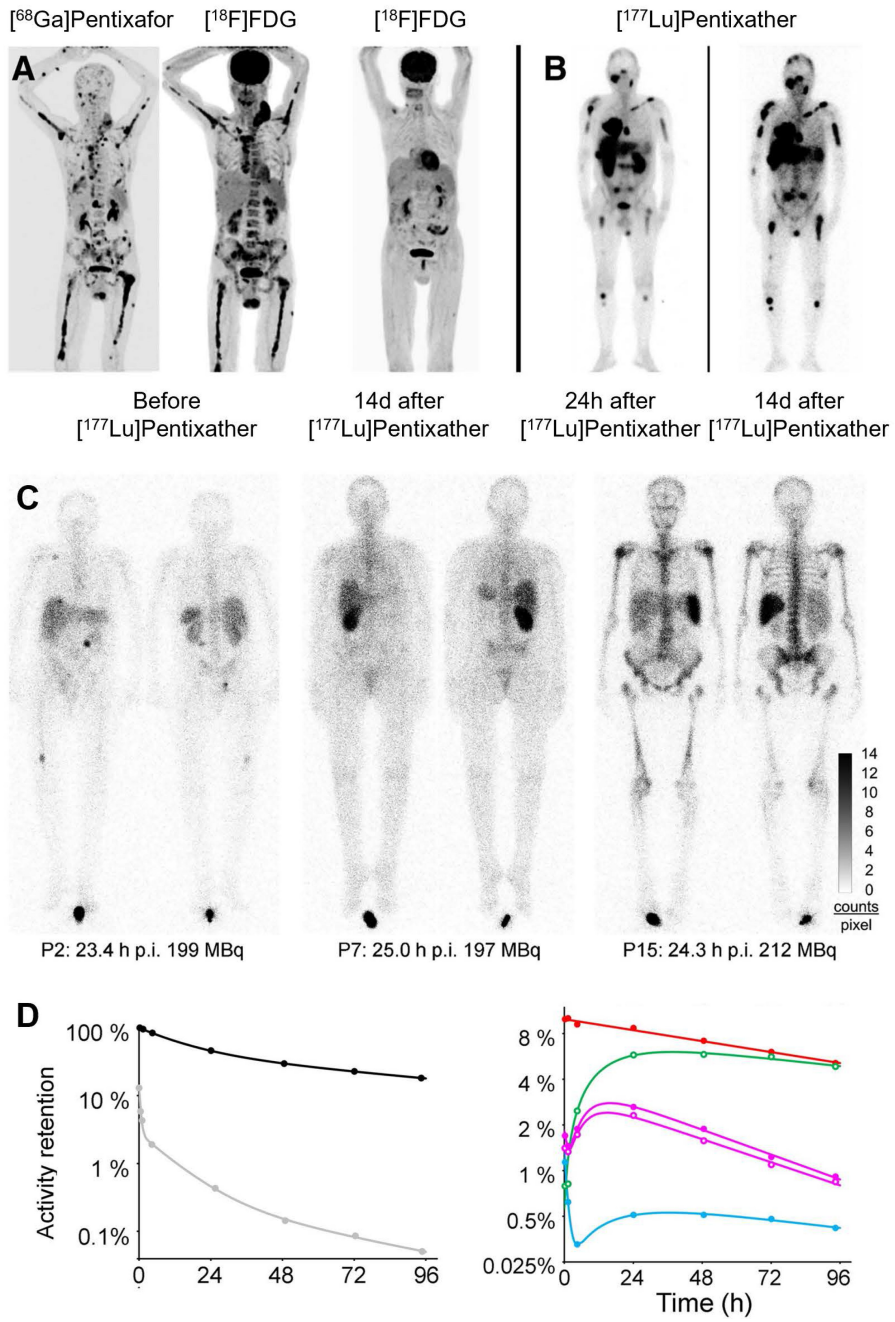
** Clinical application of [^177^Lu]/[^90^Y]Pentixather. (A)** Pre-therapy [^68^Ga]Pentixafor and [^18^F]FDG PET/CT MIP in Patient 3 show high CXCR4 expression in multiple FDG-avid extra-/intramedullary myeloma lesions. Post-therapy [^18^F]FDG PET/CT at 2 weeks after [^90^Y]Pentixather demonstrates complete metabolic response. **(B)** Scintigraphy in Patient 1 at 24 h and 15 d after 15.2 GBq [^177^Lu]Pentixather confirms CXCR4 targeting. Altered tumor-to-background ratios reflect decreased background uptake and extended acquisition times at later timepoints. Adapted with permission from 20, copyright © 2016, Society of Nuclear Medicine and Molecular Imaging. **(C)** Variable biodistribution of [^177^Lu]Pentixather in Patient 2/7 (multiple myeloma) and Patient 15 (pre-B-ALL). While renal uptake reached 5% (single kidney, P7), total renal uptake was only 1.1% in P15. In contrast, P15 showed 3-fold higher bone marrow and 11-fold higher splenic retention. **(D)** Regions of interest for time-activity curves in Patient 3: whole body (black), red marrow (red), liver (green), right/left kidneys (purple solid/open circles), spleen (blue) with fit functions. Gray symbols with fitting curve represent activity retention per liter of whole blood. Adapted with permission from 170, copyright © 2022, Society of Nuclear Medicine and Molecular Imaging.

The inaugural clinical application of [^177^Lu]/[^90^Y]Pentixather focused on CXCR4-targeted radionuclide therapy in advanced MM 20 (Figure 10A-B). Initial studies involving high-risk MM patients demonstrated promising initial response rates with favorable safety and tolerability profiles. However, these early outcomes did not translate into significant overall survival prolongation. Biokinetic and dosimetric analyses by Heribert *et al.* revealed median absorbed doses of 0.91 Gy (range: 0.38 - 3.47 Gy) to kidneys and 0.47 Gy (range: 0.14 - 2.33 Gy) to bone marrow per GBq of [^177^Lu]Pentixather, while [^90^Y]Pentixather delivered corresponding doses of 3.75 Gy (range: 1.48 - 12.2 Gy) and 1.60 Gy (range: 0.27 - 4.45 Gy) per GBq 170 (Figure 10C-D). Tumor and extramedullary lesions received estimated doses ranging from 1.5 to 18.2 Gy/GBq of [^90^Y]Pentixather. For hematologic malignancies, these calculated absorbed doses to bone marrow and extramedullary lesions may provide valuable adjunctive therapy alongside high-dose chemotherapy in stem cell transplantation protocols.

Beyond MM, CXCR4-targeted radionuclide therapy has shown potential across various hematologic and solid malignancies 90,171,172. In AML, [^177^Lu]Pentixather has been investigated as a conditioning regimen before hematopoietic stem cell transplantation, demonstrating efficient leukemia cell reduction while exhibiting acceptable toxicity profiles 172. Preliminary results indicate successful bone marrow targeting with minimal damage to vital organs, suggesting potential for improving transplantation outcomes. Due to high physiological CXCR4 expression in the bone marrow, CXCR4-targeted radionuclide therapy is associated with severe, on-target marrow toxicity that is often intentional in conditioning settings. Consequently, most clinical Pentixather-based TRT protocols have incorporated or required subsequent hematopoietic stem cell transplantation, substantially limiting broader clinical applicability.

For solid tumors, clinical experience remains limited but promising. Case reports have documented [^177^Lu]Pentixather application in CXCR4-positive GBM, pancreatic cancer, triple-negative breast cancer, and neuroendocrine cancer, where conventional therapies had been exhausted 173. These reports describe disease stabilization and symptomatic improvement in selected patients, particularly those with high CXCR4 expression confirmed by [^68^Ga]Pentixafor PET imaging. The therapy appears most beneficial for patients with limited tumor burden and predominantly osseous metastases, where favorable biodistribution patterns enhance therapeutic efficacy.

Several challenges require addressing for optimal clinical implementation. The heterogeneous expression of CXCR4 within tumors may lead to incomplete lesion targeting, while potential hematological toxicity necessitates careful patient selection and dose optimization 85,174. Current research focuses on combination strategies with conventional chemotherapy, immunotherapy, and other targeted agents to enhance treatment efficacy 172. Additionally, the development of alpha-emitting CXCR4-targeted radiopharmaceuticals (e.g., [^225^Ac]Pentixather) represents an emerging approach to improve therapeutic index, particularly for micrometastatic disease.

Ongoing clinical trials are systematically evaluating [^177^Lu]/[^90^Y]Pentixather across various malignancies, with particular emphasis on optimal dosing schedules, combination regimens, and patient selection criteria based on comprehensive CXCR4 expression profiling 8,169,171. As the clinical experience matures, CXCR4-targeted radionuclide therapy is poised to become an important component in the precision oncology arsenal, especially for malignancies characterized by CXCR4 overexpression and dissemination patterns amenable to targeted radionuclide approaches.

### 4.2. Limitations and challenges of CXCR4-targeted radionuclide therapy

While CXCR4-targeted radionuclide therapies, represented by [^177^Lu]/[^90^Y]Pentixather, have demonstrated promising initial efficacy in various hematologic malignancies and some solid tumors, it is essential to critically recognize the multiple challenges they face in clinical translation and application 20,33. The primary challenge stems from the inherent toxicity associated with the target's biological characteristics, rooted in the fundamental contradiction that CXCR4 is not tumor-specific. Its persistent and high expression on normal hematopoietic stem and progenitor cells inevitably leads to significant "off-target" bone marrow toxicity during targeted radionuclide therapy 170. This results in dose-limiting severe myelosuppression, the direct clinical consequence of which is that the vast majority of existing treatment protocols must incorporate planned autologous hematopoietic stem cell transplantation as a rescue measure 8. This not only substantially increases the complexity and risks of the treatment but also fundamentally restricts its current applicability to a select minority of patients who are in good physical condition and eligible for transplantation, introducing significant patient selection bias.

Even within tumors exhibiting high CXCR4 expression, significant intra- and inter-tumoral heterogeneity exists in expression levels 175. Molecular imaging often reveals heterogeneous tracer uptake across different lesions and even within the same lesion. This leads to uneven radiation dose distribution, creating inadequately irradiated "cold spots" that can become sources of tumor recurrence or progression 176-180. Consequently, the observed therapeutic responses are frequently characterized by limited depth of response and short duration of remission. Furthermore, therapeutic pressure may select for clones with low or negative CXCR4 expression, leading to acquired resistance, which is a key reason for the current lack of clear overall survival benefit data 181. Compared to the established roles of PSMA and SSTR2 in specific cancer types, the optimal patient population for CXCR4-targeted therapy still requires more precise definition.

## 5. Conclusion and future perspectives

This comprehensive review has delineated the remarkable progress achieved in targeting the CXCR4/CXCL12 axis for diagnostic and therapeutic purposes in oncology and beyond. The development of the high-affinity CXCR4 ligand Pentixafor and its subsequent radiolabeling with Gallium-68 have established [^68^Ga]Pentixafor PET as a powerful and versatile non-invasive tool for quantifying CXCR4 expression *in vivo*. Its robust clinical performance has been demonstrated across a remarkably wide spectrum of conditions, spanning hematologic malignancies, solid tumors, endocrine disorders, and inflammatory diseases 16,109-111,114,117,119. The ability to precisely visualize and quantify CXCR4 expression has proven invaluable for tumor subtyping, staging, prognostic stratification, and treatment response assessment, often outperforming or complementing conventional imaging modalities like [^18^F]FDG PET/CT, particularly in scenarios involving bone marrow involvement or low metabolic activity 60,64,71,84.

The successful translation of the corresponding therapeutic agent, [^177^Lu]/[^90^Y]Pentixather, has completed the radiotheranostic cycle, enabling personalized CXCR4-directed endoradiotherapy 8,172,182,183. Initial clinical applications, primarily in advanced hematologic malignancies like MM and AML, have confirmed the feasibility and safety of this approach, paving the way for its broader exploration. The paradigm of using [^68^Ga]Pentixafor for patient selection and treatment planning ensures that therapy is delivered to those most likely to benefit, thereby optimizing the therapeutic index. While the Pentixafor/Pentixather system is the leading clinical CXCR4 theranostic pair, its development benefited from earlier efforts with other agents. Initial trials using radiolabeled AMD3100 confirmed CXCR4 targeting but were limited by poor pharmacokinetics 184. Other CXCR4-targeting compounds in clinical testing often faced challenges such as low tumor-to-background ratios, high uptake in non-target organs, or stability issues. This highlights that the success of Pentixafor-based agents depends not only on CXCR4 expression but also on their optimized pharmacokinetic and binding properties.

Despite these significant advancements, several challenges and opportunities for future research remain. A primary challenge is the heterogeneity of CXCR4 expression within and between tumors, which can lead to incomplete targeting and potential treatment resistance 185. Future efforts should focus on elucidating the complex regulation of CXCR4 expression and its dynamic interplay within the tumor microenvironment. Combination therapies represent a particularly promising avenue 186,187. A positive [^68^Ga]Pentixafor PET signal reflects the aggregate CXCR4 expression from multiple cellular sources within the lesion, including malignant tumor cells, immune cell infiltration, stromal and progenitor cells in the tumor microenvironment, as well as non-specific inflammatory processes. Therefore, PET positivity should not be equated solely with tumor cell burden. This biological complexity implies that imaging positivity cannot definitively predict the therapeutic efficacy of [^177^Lu]/[^90^Y]Pentixather 172. If a significant portion of the radiation dose is delivered to non-malignant CXCR4-expressing cells, the therapeutic index may be compromised. Moreover, targeting CXCR4 on stromal or immune cells risks exacerbating on-target toxicity, particularly myelosuppression, without proportionally enhancing tumor cell kill. Consequently, complementary diagnostic methods, such as immunohistochemistry or spatial transcriptomic analysis of biopsy samples, are necessary to deconvolute the cellular origins of CXCR4 expression and better identify patients most likely to benefit from CXCR4-targeted theranostics.

Integrating CXCR4-targeted radionuclide therapy with conventional chemotherapy, immunotherapy, external beam radiotherapy, or other targeted agents could yield synergistic effects by overcoming resistance mechanisms and modulating the immune landscape. For instance, combining CXCR4 inhibition with immune checkpoint blockers could potentially reverse immunosuppression and enhance anti-tumor immunity. The evolution of theranostic agents themselves is another critical frontier 188. The development of alpha-emitter-labeled Pentixather analogs, such as [^225^Ac]Pentixather, holds great promise for further improving efficacy, especially against micrometastatic disease, due to the higher linear energy transfer and shorter tissue penetration of alpha particles, which may potentiate greater cytotoxic effects while potentially sparing surrounding healthy tissues. However, when targeting CXCR4, alpha-emitters carry an unacceptable risk of bone marrow toxicity. The high LET radiation renders the bone marrow highly vulnerable, and currently, there is a lack of feasible strategies to overcome this challenge. Consequently, their clinical feasibility remains highly uncertain and requires further validation. Furthermore, the application of artificial intelligence and radiomics to [^68^Ga]Pentixafor PET imaging data could unlock deeper insights. These approaches may enable the extraction of sub-visual features predictive of treatment response, patient prognosis, and CXCR4 signaling pathway activity, moving towards even more refined precision medicine.

In conclusion, CXCR4-targeted theranostics, exemplified by the [^68^Ga]Pentixafor /[^177^Lu]Pentixather pair, has firmly established itself as a transformative strategy in modern medicine. It embodies the core principle of precision medicine, "see what you treat, treat what you see." As ongoing research continues to address current limitations and explore new combinations and technological advancements, CXCR4-directed theranostics is poised to expand its impact, offering renewed hope for patients with a diverse range of CXCR4-driven diseases and solidifying its role as a cornerstone of targeted radionuclide therapy.

## Figures and Tables

**Table 1:** Summary of key clinical studies evaluating [^68^Ga]Pentixafor PET across disease entities.

Authors	Year	Population	Number of patients	Method	Ref
Herrmann *et al.*	2015	MM	5	Prospective	31
Philipp-Abbrederis *et al.*	2015	MM	14	Prospective	32
Wang *et al.*	2015	GBM	8	Prospective	36
Reiter *et al.*	2015	MI	7	Prospective	37
Vag *et al.*	2016	Solid cancers	21	Prospective	38
Lapa *et al.*	2016	SCLC	10	Prospective	39
Lapa *et al.*	2016	GBM	15	Prospective	40
Herhaus *et al.*	2016	AML	10	Prospective	41
Bluemel *et al.*	2017	Adrenocortical cancer	30	Prospective	42
Lapa *et al.*	2017	MM	35	Prospective	43
Derlin *et al.*	2017	UTI	13	Prospective	44
Werner *et al.*	2017	Neuroendocrine tumors	12	Prospective	45
Herhaus *et al.*	2017	MZL	1	Retrospective	46
Lapa *et al.*	2017	Pleural mesothelioma	6	Retrospective	47
Bouter *et al.*	2018	Chronic Bone Infection	14	Prospective	48
Weiberg *et al.*	2018	AP	51	Retrospective	49
Li *et al.*	2018	AP	38	Prospective	50
Mayerhoefer *et al.*	2018	CLL	23	Prospective	51
Derlin *et al.*	2018	AP	37	Prospective	52
Bouter *et al.*	2018	Chronic osteomyelitis	29	Retrospective	53
Li *et al.*	2019	AP	72	Prospective	54
Luo *et al.*	2019	WM/LPL	17	Prospective	55
Breun *et al.*	2019	Vestibular schwannomas	4	Prospective	56
Haug *et al.*	2019	MALT	36	Prospective	57
Werner *et al.*	2019	Solid tumors	19	Retrospective	58
Kircher *et al.*	2020	AP	92	Retrospective	59
Pan *et al.*	2020	MM	30	Prospective	60
Ding *et al.*	2020	PA	36	Prospective	61
Herhaus *et al.*	2020	CNSL	11	Prospective	62
Pan *et al.*	2020	Non-Hodgkin lymphoma	27	Retrospective	63
Lawal *et al.*	2020	AP	12	Prospective	64
Starzer *et al.*	2021	CNSL	7	Prospective	65
Mayerhoefer *et al.*	2021	MCL	22	Prospective	66
Linde *et al.*	2021	NEC	10	Retrospective	67
Duell *et al.*	2021	MZL	22	Retrospective	68
Weich *et al.*	2021	NEC	11	Retrospective	69
Werner *et al.*	2021	MI	96	Retrospective	70
Pan *et al.*	2021	WM/LPL	15	Prospective	71
Kuyumcu *et al.*	2021	MM	24	Retrospective	72
Lewis *et al.*	2021	Solid cancer	145	Retrospective	73
Sarah *et al.*	2022	GBM	7	Retrospective	74
Kraus *et al.*	2022	MPNs	12	Retrospective	75
Mayerhoefer *et al.*	2022	MALT	26	Prospective	76
Chen *et al.*	2022	CNSL	26	Prospective	77
Kwon *et al.*	2022	MCL	146	Retrospective	78
Buck *et al.*	2022	Solid or hematologic neoplasms	690	Retrospective	79
Serfling *et al.*	2022	Solid tumors	90	Retrospective	80
Ding *et al.*	2022	Cushing syndrome	31	Retrospective	81
Watts *et al.*	2022	Rare lung malignancies	6	Prospective	82
Shekhawat *et al.*	2022	MM	34	Prospective	83
Lu *et al.*	2022	AP	19	Retrospective	84
Kraus *et al.*	2022	MM	87	Retrospective	85
Gao *et al.*	2023	PA	50	Prospective	86
Watts *et al.*	2023	Lung cancer	94	Prospective	87
Mayerhoefer *et al.*	2023	MCL	16	Prospective	88
Kosmala *et al.*	2023	MZL	73	Retrospective	89
Hartlapp *et al.*	2023	DSRCT	8	Prospective	90
Roustaei *et al.*	2023	GBM	24	Prospective	91
Zheng *et al.*	2023	PA	120	Prospective	92
Zhi *et al.*	2023	HNSCC	12	Retrospective	93
Kosmala *et al.*	2024	MZL	32	Retrospective	94
Dreher *et al.*	2024	Solid tumors	142	Retrospective	95
Jena *et al.*	2024	Soft tissue/bone sarcoma	10	Prospective	96
Waheed *et al.*	2024	GBM	19	Prospective	97
Yin *et al.*	2024	PA	19	Prospective	98
Liu *et al.*	2024	NPC	25	Prospective	99
Pan *et al.*	2024	sWM	48	Retrospective	100
Schloetelburg *et al.*	2024	ACC	41	Retrospective	101
Hadebe *et al.*	2024	HNSCC	23	Prospective	102
Chen *et al.*	2024	IBD	5	Retrospective	17
Roustaei *et al.*	2024	GBM	26	Prospective	103
Kopp *et al.*	2024	SSc-ILD	22	Prospective	104
Wu *et al.*	2024	Cushing Disease	43	Retrospective	105
Yang *et al.*	2024	MM	19	Prospective	106
Zuo *et al.*	2025	PA	61	Retrospective	107
Yi *et al.*	2025	PA	37	Prospective	108
Zhang *et al.*	2025	PA	208	Retrospective	109
Chen *et al.*	2025	CNSL\GBM	124	Retrospective	110
Kosmala *et al.*	2025	AP	65	Retrospective	111
Li *et al.*	2025	PA	27	Retrospective	112
Zheng *et al.*	2025	PA	91	Prospective	113
Wang *et al.*	2025	Thymoma	32	Prospective	114
Kaur *et al.*	2025	MM	40	Prospective	115
Gauthaman *et al.*	2025	MM	13	Prospective	116
Hadebe *et al.*	2025	Breast cancer	51	Prospective	117
Zheng *et al.*	2025	PA	90	Prospective	118
Meng *et al.*	2025	PA	62	Prospective	119
Pan *et al.*	2025	MM	25	Retrospective	120
Zhoufei *et al.*	2025	PA	25	Prospective	121
Shu *et al.*	2025	PA	51	Retrospective	122
Zheng et al	2025	PA	197	Prospective	123
Diekmann *et al.*	2025	MI	49	Retrospective	16

MM: multiple myeloma; GBM: glioblastoma; MI: myocardial infarction; SCLC: small cell lung cancer; AML: acute myeloid leukemia; UTI: urinary tract infection; MZL: marginal zone lymphoma; AP: atherosclerotic plaque; CLL: chronic lymphocytic leukemia; WM/LPL: waldenström macroglobulinemia/lymphoplasmacytic lymphoma; MALT: mucosa-associated lymphoid tissue; PA: primary aldosteronism; CNSL: central nervous system lymphoma; MCL: mantle cell lymphoma; NEC: esophageal cancer; MPNs: myeloproliferative neoplasms; DSRCT: desmoplastic small round cell tumor; NPC: nasopharyngeal carcinoma; sWM: smoldering waldenström macroglobulinemia; ACC: adrenal cortical carcinoma; HNSCC: head and neck squamous cell carcinoma; IBD: inflammatory bowel disease; SSc-ILD: systemic sclerosis-associated interstitial lung disease.

**Table 2:** Summary of key clinical studies evaluating [^177^Lu]/[^90^Y]Pentixather across disease entities.

Authors	Year	Population	Number of patients	Radiopharmaceutical	Ref
Herrmann *et al.*	2016	MM	3	[^177^Lu]/[^90^Y]Pentixather	20
Habringer *et al.*	2018	AML	3	[^177^Lu]Pentixather	33
Lapa *et al.*	2018	MM	3	[^177^Lu]Pentixather	167
Maurer *et al.*	2019	Lymphoproliferative or myeloid malignancies	22	[^177^Lu]/[^90^Y]Pentixather	168
Lapa *et al.*	2019	DLBCL	6	[^177^Lu]/[^90^Y]Pentixather	169
Hänscheid *et al.*	2022	Hematological malignancies	19	[^177^Lu]/[^90^Y]Pentixather	170
Hartlapp *et al.*	2023	DSRCT	8	[^90^Y]Pentixather	90
Dreher *et al.*	2024	Hematological malignancies	21	[^90^Y]Pentixather	171
Braitsch *et al.*	2025	AML	7	[^177^Lu]Pentixather	172
Dreher *et al.*	2025	MM	38	[^177^Lu]/[^90^Y]Pentixather	8

MM: multiple myeloma; AML: acute myeloid leukemia; DLBCL: diffuse large B-cell lymphoma; DSRCT: Desmoplastic small round cell tumors.

## Abbreviations

HIV-1: human immunodeficiency virus type 1
MM: multiple myeloma
AML: acute myeloid leukemia
PET: positron emission tomography
SCLC: small cell lung cancer
TBR: tumor-to-background ratio
AMI: acute myocardial infarction
PA: primary aldosteronism
APA: aldosterone-producing adenoma
IHA: idiopathic hyperaldosteronism
NFA: non-functioning adrenal adenomas
CT: computed tomography
SUV: standardized uptake value
ACTH: adrenocorticotropic hormone
MRI: magnetic resonance imaging
MZL: marginal zone lymphoma
WM/LPL: waldenström macroglobulinemia/lymphoplasmacytic lymphoma
MCL: mantle cell lymphoma
CNSL: central nervous system lymphoma
CLL: chronic lymphocytic leukemia
MALT: mucosa-associated lymphoid tissue
GBM: glioblastoma
R-CT: radiochemotherapy
HNSCC: head and neck squamous cell carcinoma
NPC: nasopharyngeal carcinoma
NSCLC: non-small cell lung cancer
ACC: adrenal cortical carcinoma
